# An Overview of Cellulose Derivatives-Based Dressings for Wound-Healing Management

**DOI:** 10.3390/ph14121215

**Published:** 2021-11-24

**Authors:** Elena-Emilia Tudoroiu, Cristina-Elena Dinu-Pîrvu, Mădălina Georgiana Albu Kaya, Lăcrămioara Popa, Valentina Anuța, Răzvan Mihai Prisada, Mihaela Violeta Ghica

**Affiliations:** 1 Department of Physical and Colloidal Chemistry, Faculty of Pharmacy, Carol Davila University of Medicine and Pharmacy Bucharest, 6 Traian Vuia Str., 020956 Bucharest, Romania; elena-emilia.tudoroiu@drd.umfcd.ro (E.-E.T.); lacramioara.popa@umfcd.ro (L.P.); valentina.anuta@umfcd.ro (V.A.); razvan.prisada@umfcd.ro (R.M.P.); mihaela.ghica@umfcd.ro (M.V.G.); 2 Department of Collagen, Division Leather and Footwear Research Institute, National Research and Development Institute for Textile and Leather, 93 Ion Minulescu Str., 031215 Bucharest, Romania

**Keywords:** cellulose derivatives, wound dressings, wound management, bioactive agents delivery systems

## Abstract

Presently, notwithstanding the progress regarding wound-healing management, the treatment of the majority of skin lesions still represents a serious challenge for biomedical and pharmaceutical industries. Thus, the attention of the researchers has turned to the development of novel materials based on cellulose derivatives. Cellulose derivatives are semi-synthetic biopolymers, which exhibit high solubility in water and represent an advantageous alternative to water-insoluble cellulose. These biopolymers possess excellent properties, such as biocompatibility, biodegradability, sustainability, non-toxicity, non-immunogenicity, thermo-gelling behavior, mechanical strength, abundance, low costs, antibacterial effect, and high hydrophilicity. They have an efficient ability to absorb and retain a large quantity of wound exudates in the interstitial sites of their networks and can maintain optimal local moisture. Cellulose derivatives also represent a proper scaffold to incorporate various bioactive agents with beneficial therapeutic effects on skin tissue restoration. Due to these suitable and versatile characteristics, cellulose derivatives are attractive and captivating materials for wound-healing applications. This review presents an extensive overview of recent research regarding promising cellulose derivatives-based materials for the development of multiple biomedical and pharmaceutical applications, such as wound dressings, drug delivery devices, and tissue engineering.

## 1. Introduction

Skin is the largest and the main organ that forms the body covering, with a complex structure of tissues, and creates an exterior defense barrier, which protects the internal organs from mechanical impairments, radiation, chemicals, and foreign invaders (bacteria and viruses) [1]. More than being a passive barrier, the skin defends the body against contamination, infection, and surrounding environment influence [2]. Skin is also a sensory organ and contains a large category of sensory neuron subtypes (thermoreceptors, nociceptors, pruriceptors, and low-threshold mechanoreceptors), that take over and transfer to the brain information about the environment [3]. Moreover, the skin has an important role in homeostasis, elimination of toxins, sustaining regular hydration levels, prevention of electrolytes loss [4], and in control of body temperature and blood pressure. The skin is made up of three particular layers: epidermis, dermis, and hypodermis or subcutaneous layer [5].

The epidermis is a physical protective barrier against the external factors, which does not contain blood vessels. It is comprised of two main categories of cells: dendritic cells and keratinocytes (keratin synthesis), along with Langerhans cells (engaged in the immune reaction), Merkel cells (sensory corpuscles), and melanocytes (melanin synthesis) [6,7]. The dermis is situated between the epidermis and hypodermis, and it is responsible for skin thickness. The dermis structure is principally fibrous because it contains collagen and elastic fibers [8]. Moreover, this layer also includes hair follicles, sebaceous glands, sweat glands, nerves, and blood vessels. The fundamental component of the dermis is collagen and the most abundant are type I and type III (approximately 95%) [9,10]. The dermis plays an essential role in thermoregulation, skin protection, preservation of skin support, and perception of sensation [11]. The hypodermis (subcutaneous tissue), the widest and the thickest part of the skin, is located between the dermis and muscles or bones beneath it and it is made up of elastin and loose connective tissue [12]. The principal roles of subcutaneous tissue are thermal insulation, energy resource, nutritional reserve, and mechanical conservation [13,14].

There are many factors and systemic diseases that can alter skin functions, for example, pathogens, pollution, radiation exposure, smoking, malnutrition, obesity, diabetes mellitus, peripheral vascular diseases, pressure ulcer, inflammatory, bleeding, or metabolic conditions and immunosuppression [15,16]. In many cases, all these factors can delay wound healing, with harmful risks for patients, such as oxidative stress, chronic inflammation, infection, increased tissue loss, and necrosis [17]. Thus, skin tissue disorders are a public health problem worldwide, with a higher incidence from year to year. For example, in 2005 there were estimated ~5 million skin and subcutaneous conditions, in 2015 ~6.1 million [18], and in 2018 there were ~8.2 million people, who suffered from wounds. Total costs of treatments for wound healing vary between $28.1 billion and $96.8 billion [19]. With a prevalence of 1–2% in the global population, chronic wounds have the largest frequency from all types of skin tissue injuries, especially surgical wounds, and leg/foot ulcers (pressure ulcer and diabetic foot ulcer) [20]. An injury involves physical impact, such as pain, inflammation, mobility limitation, disturbance of sleep, alterations of skin appearance, and restriction of daily activities; consequently, these effects have a negative impact on the patient quality of life, affecting emotional, social, and physical states [21]. To restore the impaired tissue and to rebalance the quality of life for patients with wounds, optimal and multidisciplinary wound management has an essential role. Its main purpose is to obtain a proper functional, structural, and cosmetic result [22]. Frequently, to alleviate the pain and inflammation, which accompany the wounds, analgesic and anti-inflammatory drugs are prescribed. Because of their side effects on the gastrointestinal system when they are administered orally [23], a more advantageous and simple treatment is to apply on the lesion site a wound dressing [24]. The main purpose of wound management is to reduce the period of wound healing through the prevention of infection, alleviation of inflammation and pain, and diminishing the scars [25].

In this review, we mainly present studies from the last 10 years regarding the cellulose derivatives-based wound dressings with various bioactive agents to accelerate the regeneration of skin tissue injuries. Cellulose derivatives have proper and optimal applicability to develop novel wound dressings that can enhance wound healing, obtained by substituting the hydroxyl groups from cellulose molecule with different alkyl groups [26]. Thus, they exhibit high solubility in water and represent a feasible alternative to water-insoluble cellulose. Moreover, these semi-synthetic biopolymers possess other multiple characteristics, such as biocompatibility, biodegradability, proper physicochemical properties, low toxicity and costs, poor immunogenicity, adequate absorption of wound exudates, thermo-gelling power, and antibacterial effect [27,28]. Besides wound-dressings development, cellulose derivatives are promising biomaterials for tissue engineering, drug delivery, hemodialysis, osseointegration, and biosensors [29].

## 2. Wound Classification

A wound represents a lesion, break, tear, or damage of skin structure and function, produced by physical, mechanical (surgery), thermal, chemical, and electrical (burns) factors; an injury can also be the result of an underlying medical or physiological disorder (diabetes and malignancies) [30,31,32]. The Wound Healing Society define a wound as a consequence of ‘disruption of normal anatomic structure and function’ [33]. The National Institutes of Health from the United States assesses that approximately 3% of people over the age of 65 can suffer at any one time from a cutaneous lesion [34].

Wounds are classified according to many parameters:

- etiology: surgical, traumatic, radiation and malignant wound, chemical or thermal injury, pressure ulcer, diabetic foot ulcer, vascular ulcer, or atypical injury;
- healing time (duration) and nature of the injury restoration process: acute or chronic wounds;
- depth of injury or number of skin layers affected: superficial, partial thickness (deep dermal) or full-thickness wounds;
- complexity: simple, complex, or complicated wounds;
- contamination and postoperative infection risk: clean wound (class I), clean/contaminated wound (class II), contaminated wound (class III) or dirty wound (class IV);
- mode of lesion: abrasion, ulceration, incision, laceration or degloving;
- tissue loss: without tissue loss (surgical wounds) and with tissue loss (burns, traumatic wounds, diabetic foot ulcers, and iatrogenic wounds);
- appearance: necrotic, sloughy, infected, malodorous, granulating, and epithelializing wound;
- injured tissue coloration: black, green, yellow, white, brown, purple, beefy red, or pale pink wounds [35,36,37].

From all of the classification criteria, the most significant and decisive criterion for selection of an adequate dressing and for optimal wound-healing management is the healing time (duration) and the nature of the injury restoration process. Hence, an acute injury heals totally, without external support, with minimum scarring, and usually demands a period for healing from 8 to 12 weeks [38]. An acute lesion can be simple or complex, but it depends on the affected anatomical parts and on the dimension and depth of this lesion. In this category are found mechanical injuries, burns, and chemical wounds [33,39]. In contrast to the acute wound, a chronic injury heals slowly, requires a long time for healing, more than 12 weeks, usually reoccurs, and leaves severe scars; mostly, a chronic wound does not have any time limitation for the repair process [40]. The main chronic wounds are venous ulcer, ischemic injuries (especially of atherosclerotic origin), diabetic foot ulcer, pressure ulcer, and malignant wounds [41,42].

## 3. Wound-Healing Process

Wound healing is a sophisticated and well-coordinated process [43], which involves a variety of cellular and biochemical reactions that need a complex and dynamic cascade of biological processes [44,45] for the reestablishment of skin layers, growth and tissue regeneration, anatomical continuity, and skin functions [46]. Damaged skin tissue has the capacity to repair itself to form a new epithelium that closes the wound and repairs the barrier function, through an intricate process [47]. 

### 3.1. Wound-Healing Stages

**Figure 1:**
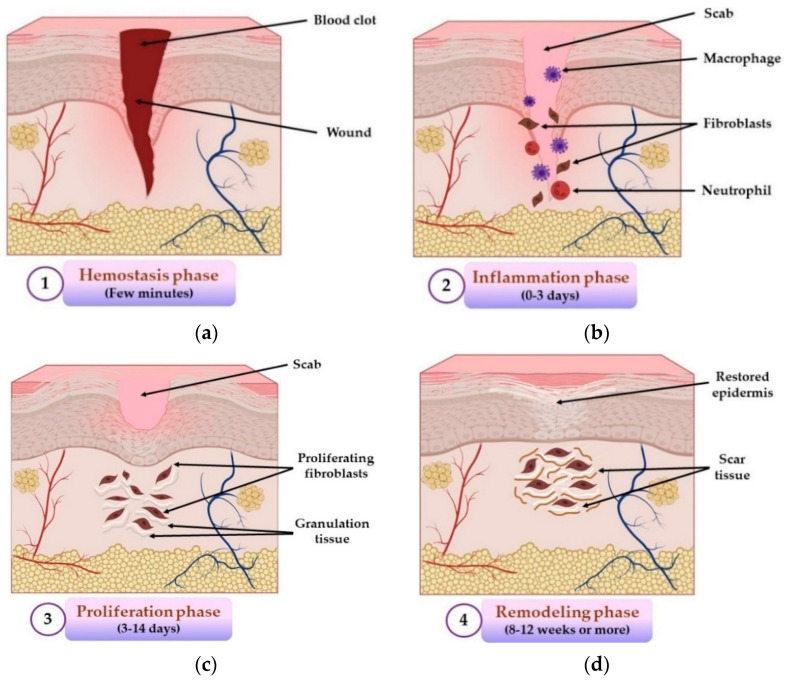
The four stages of wound healing process: (**a**) hemostasis phase; (**b**) inflammation phase; (**c**) proliferation phase; (**d**) remodeling phase. All illustrations have been created with BioRender.com, Inkscape, and PowerPoint.

The wound repair process consists of four different, overlapping, and exactly programmed stages: hemostasis, inflammation, proliferation, and remodeling (maturation) phases [48], illustrated in Figure 1.

The first and the shortest stage (5–10 min) of the wound repair process is hemostasis, an instantaneous reaction towards lesion [49], whose main purpose is to stop the bleeding through vasoconstriction, primary hemostasis (thrombocytes aggregation with thrombocyte plug formation) and secondary hemostasis (fibrin clot formation) [50]. The inflammation stage happens approximately at the same time as the hemostatic stage and consists of enrollment of neutrophils and macrophages, cytokines secretion, destruction and elimination of bacteria and formation of a wound bed [51]. Inflammation induces accumulation of leukocytes at the lesion’s level, activating different mediators and chemotactic factors in 1–2 days after injury and lasts for about 3 days [47]. The proliferation stage begins on day 3 and can have a duration up to 14 days after tissue damage [52]. This phase represents a complex process that includes the next events: neoangiogenesis, production of granulation tissue through fibroblasts proliferation and collagen deposition, synthesis of extracellular matrix, re-epithelialization, and injury retirement, all these happening simultaneously [53]. The final phase of the wound repair process is the remodeling (maturation) stage, and its major purpose is the production of cellular connective tissue and hardening of the new epithelium that establishes the final scar nature [54]. It is the longest stage of all five and can last from weeks to 1–2 years or more. The main event of this phase is the remodeling of granulation tissue, where collagen type I will take the place of collagen type III because type I is more stable [55]. 

### 3.2. Factors Affecting Wound-Healing Process

**Figure 2:**
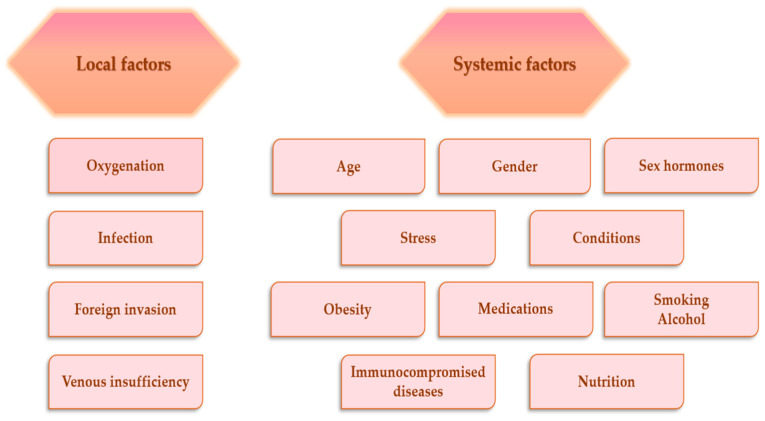
Factors affecting wound-healing process [57,58].

Many factors can interfere with the wound-healing phases, the consequence being an improper or damaged wound repair process. In general, these factors can be categorized as local and systemic. Local factors affect features of the lesion itself, and systemic factors represent the general health or condition states of one person, which influence the capacity to heal [56]. The main factors that affect the wound-healing process are presented in Figure 2.

#### 3.2.1. Local Factors That Affect Wound-Healing Process

In injured tissues, vascular disruption generates depletion of oxygen, causing hypoxia, and, consequently, impaired wound healing. Temporary hypoxia stimulates the lesion repair process, but persistent hypoxia prolongs this process [59]. Hypoxia is characterized by high levels of reactive oxygen species (ROS) in cells and the impact on tissue healing is deleterious [31,57]. Oxygen has many roles in the injured tissue: avoids infection, activates the angiogenesis, enhances keratinocytes differentiation, movement, and re-epithelialization, increases fibroblast proliferation and biosynthesis of collagen, and stimulates lesion contraction [60]. When skin tissues are injured, the physical protective barrier against foreign invaders is damaged, these germs easily invade the lesion and contaminate or colonize it, causing local infection, and in severe cases, when the injury is not treated properly, they cause systemic infection [61]. Moreover, bacteria and endotoxins may induce the extended elevation of matrix metalloproteinases and pro-inflammatory cytokines (IL-1 and TNF-α), prolonging the inflammatory phase [56]. 

#### 3.2.2. Systemic Factors That Affect Wound-Healing Process

A main risk factor for the damaged lesion repair process is increased age due to multiple comorbidities [62]. Acute injuries have a prolonged healing time for elderly males compared to elderly females. This fact can be explained through sex hormones, which have an essential role in the wound repair process [63]. Alongside them, stress causes the decrease of pro-inflammatory cytokine levels and the reduction of chemo-attractants expression, which are involved in the inflammation stage of wound healing [64]. Regarding the conditions, the major disease, which strongly and negatively influences the wound-healing process, is diabetes mellitus, because of the diabetic foot ulcer, which causes hypoxia, inhibition of the expansion of macrophages and neutrophils and reduction of fibroblasts proliferation [65]. Obesity represents another major factor that affects the normal repair process, because it is characterized by an augmented workload of the heart to provide oxygenated blood to skin tissues, it cannot perfuse them, the outcome being the onset of ischemia and a higher risk to develop infections [66]. Among medications, steroids and chemotherapeutic drugs can lead to delayed healing. Mechanisms through steroids that affect the wound healing are the inhibition of lesion contraction, and fibroblasts proliferation, the decrease of tensile strength, and collagen production [67]. Chemotherapeutic drugs disturb the proliferative stage through slowing cells’ movement to the lesion, angiogenesis inhibition, reduction of biosynthesis of collagen and decrease of fibrin deposition [68]. The quality and rate of the normal repair process can be also altered by smoking and alcohol because they lead to lesion infection and dehiscence, reduction of neutrophils, lessening of angiogenesis, inhibition of epithelial reconstruction and lesion contraction, and in severe cases, to necrosis of tissues [69]. Poor nutrition slows the lesion repair process through inflammation extension, inhibition of fibroblasts functions, decline of angiogenesis, and reduction of collagen biosynthesis and deposition [70]. 

## 4. Wound Dressings: Properties and Classification 

In past years, due to the technology’s noteworthy progress, various wound dressings were formulated worldwide to cure all types of tissue lesion. Dressings play a fundamental role in wound-healing management because these protect tissue lesions from external invasion (wound dressings are permeable for oxygen and moisture and function as physical barriers) [71], preventing the infection on the wound site [72]. Moreover, dressings contribute to the regeneration and restoration of epidermis and dermis layers [73,74]. 

### 4.1. Wound Dressing Properties

**Figure 3:**
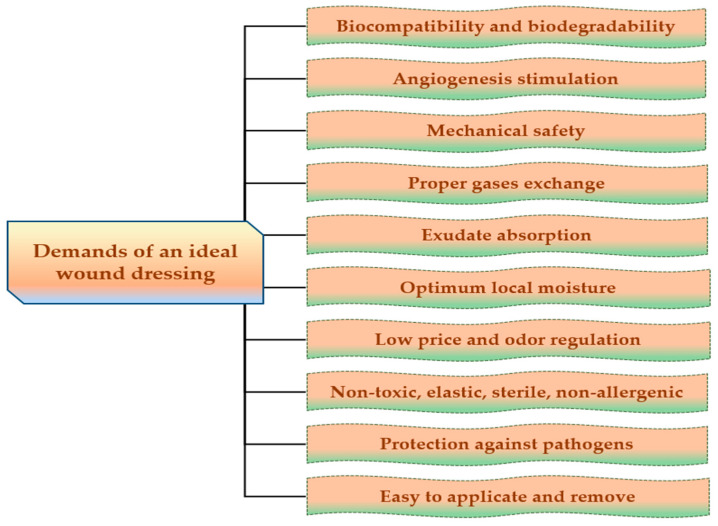
Major demands of an ideal wound dressing.

For the development of dressings, which allow rapid healing, with minimal scars on the body surface, it is necessary to develop new biopolymeric materials that accomplish some properties to create the ideal wound dressing that are reviewed in Figure 3. 

The ideal wound dressing preferably presents the following features: biocompatibility, biodegradability, non-toxicity, chemical inertness [75], to be applied effortlessly, to have the capacity to keep local moisture, to ensure a suitable exchange of gases (O_2_ and CO_2_), to absorb exudates that form on the lesion site [76], to stimulate the angiogenesis, to protect against extraneous pathogens, to clear the injured tissue, to eliminate nonviable tissues, to reduce the exposed area [77], to be able to be removed and replaced without difficulty [78], to adjust the odor, to sustain an adequate temperature to the lesion bed, to promote the blood circulation, and to stimulate cell expansion, to ensure mechanical safety [79]. Also, wound dressings materials must be elastic, sterile, non-adherent, non-allergenic [80], to have an acceptable price and to provide thermal insulation [81]. 

### 4.2. Wound-Dressing Classification

A potential classification of wound dressings comprises passive dressings and active dressings, depending on the presence or absence of one or more pharmacologically active substances or natural substances [82], which can act to the site of the lesion, with local or systemic action, conditioned by the depth of the wound. Moreover, the progress of manufacturing led to the evolution of wound dressings from traditional dressings to modern (advanced) dressings [83]. 

Passive dressings can be considered dry traditional dressings, which are fundamental for a faster wound-healing process. There is a wide simple range of passive dressings for several types of skin lesion: cotton wool, lint, gauze, natural, and synthetic bandages–they work as primary dressing or secondary dressing [79,84]. Active dressings contain a large variety of pharmacologically active substances (antibiotics or other antimicrobials, non-steroidal anti-inflammatory, analgesic, antifungal, and local anesthetics drugs) or natural substances (plant extracts) with anti-inflammatory, astringent, emollient, epithelializing, antioxidant, demulcent, and antimicrobial properties [73]. 

**Figure 4:**
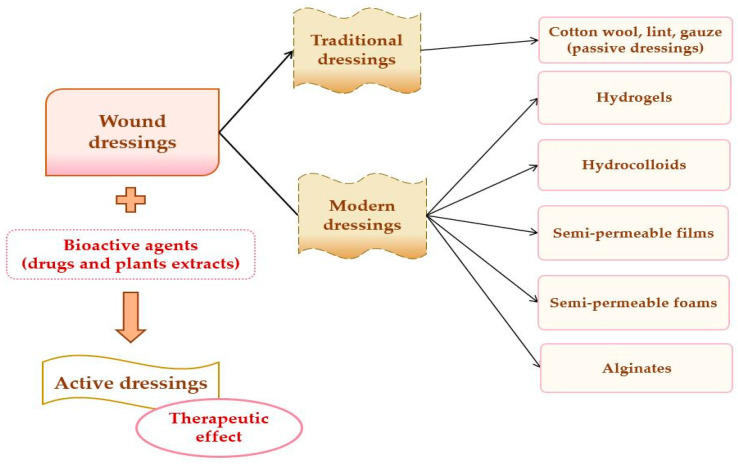
Wound dressings classification.

Modern or advanced dressings were designed to cover tissue lesions and in this category are included the hydrogels, hydrocolloids, semi-permeable films, semi-permeable foams, and alginate dressings [52,85]. The biggest difference between traditional and modern dressings is local moisture maintenance. Thus, traditional dressings have a lower capacity to maintain the local moisture on the wound site [83], and modern dressings sustain excellent local moisture to enhance wound healing [86]. The classification of wound dressings is illustrated in Figure 4.

The main materials underlying the modern wound dressings are polymers, which can be natural (collagen, gelatin, cellulose, hemicellulose, chitin, chitosan, pectins, gums, chondroitin sulfate, alginic acid, alginates, agar, dextran, carrageenan, elastin, hyaluronic acid, silk fibroin, fibrinogen, and fibrin) [87,88], semi-synthetic (cellulose derivatives) [89] or synthetic (poly(α-ester)s, polyanhydrides, polycarbonates, poly(amide), poly(esteramide)s, polyphosphazenes, polyurethanes, pseudo poly(amino acids), polyacetals) [90,91,92].

The first class of modern wound dressings includes hydrogels, also called hungry or smart networks, which can be defined as three-dimensional networks, consisting of cross-linked polymeric materials [93], with a significant capacity to absorb inside their structure a massive volume of water or body fluids, without dissolution in these liquids [94]. Hydrogels are transparent dressings, and this fact allows the tissue lesion to be observed and controlled without the dressing being eliminated [95]. Hydrogels dressings have a remarkable application in many domains due to their high water content (up to 96%) [96], such as biomedical and pharmaceutical sciences (wound dressings, drug delivery systems, diagnostics, tissue engineering, contact lenses, regenerative medicines) [97], agriculture, food industry, biotechnology, separation technology (cells and biomolecules), electroconductive hydrogels and biosensors, oil recovery, the cosmetic industry, and hygienic products [98,99,100]. Hydrogels, which stimulate autolytic debridement, are used as wound dressings in pressure ulcers, thermal injuries, and lesions caused by surgery [101].

Hydrocolloids are another class of modern wound dressings, which are based on a combination between elastomers, alginates, and colloidal materials. They present the ability to take in a small or medium quantity of exudates, have good biocompatibility, biodegradability, and adhere to the skin [102]. These dressings are occlusive, so they do not allow microorganisms to penetrate tissue lesions, do not afford gases exchanges, and are water-resistant [103]. 

The third class of modern wound dressing includes semi-permeable films. They are flexible and elastic sheets, made from transparent polyurethane. For a good attachment to the skin, polyurethane films present an acrylic adhesive on one part [104,105]. Films are impermeable to pathogens and water but allow the exchange of oxygen and water vapor. Films are used in surgical injuries or wounds with a reduced volume of exudates [106]. They cannot be applied on tissue lesions with necrosis or infection, on sensitive skin (newborns and elder people), and also on wounds, which have a substantial amount of exudates because films offer a poor capacity to absorb wound fluids [41,96].

The following category of modern dressing includes semi-permeable foams, which showed a vast improvement and favorable biocompatibility [107]. They have a considerable capacity to swallow a great quantity of liquids formed at the injury site, due to their content of hydrophilic polyurethane and silicone, so they can be recommended for tissue lesions with a medium to high status of wound fluids [108]. The disadvantages of foams consist in the limitation of use for dry wounds (foams have a dehydration effect) and in the impossibility to follow the evolution of wound healing because foams are totally opaque [109].

The last class of modern wound dressings is represented by alginates, a category of polysaccharides extracted from brown algae and kelp, with remarkable absorption properties [109]. Alginates are the result of the alginic acid reaction with calcium and sodium; therefore, alginates are called salts [50,110]. The formation of alginate gel is based on the exchange of calcium ions, which are inside the dressing, with sodium ions, which are in lesion exudates. Thus, the alginate gel presents an exceptional power to absorb a large volume of wound fluids, especially in the case of foot ulcers [111]. Another advantage of alginates dressings is that they have a hemostatic effect due to calcium ions (known as clotting factor IV, which plays an important role in blood coagulation); accordingly, they can be used if an injury bleeds [112]. 

## 5. Cellulose Derivatives as Wound Dressings

During the last decades, cellulose derivatives, also known as cellulosics, have become extensively used in many fields, from food, cosmetics, biomedical, and pharmaceutical industry [113] to biofuels and oilfield industry (petrochemicals) [114]. These semi-synthetic biopolymers present many advantageous characteristics, such as biocompatibility, biodegradability, non-toxicity, sustainability, abundance, and a suitable price; therefore, cellulose derivatives represent the first option for wound dressings development [115,116]. 

### 5.1. Cellulose Derivatives Classification

Cellulose, discovered by Anselme Payen in the 19th century, is a natural polymer, an organic polysaccharide from plant origin, non-toxic, with a structural role, being the most plentiful and renewable biopolymer on Earth [117]. Structurally, cellulose is a linear macromolecule composed of many molecules of D-glucose (the number of the glucose units can reach more than ten thousand), which are bound through 1-4-β-glycosidic linkages and its chemical formula is (C_6_H_10_O_5_)_n_ [118]. The chemical structure of cellulose shows the presence of free hydroxyl groups at C_2_, C_3_, and C_6_ of each molecule of glucose, which have a good capacity to form powerful inter- and intramolecular hydrogen bonds [119]. As a result of this property, cellulose has a crystalline and stiff structure and, consequently, it is insoluble in water and the majority of the organic solvents; moreover, this natural biopolymer cannot be digested by the human digestive system [120,121]. Cellulose has good stability to pH fluctuations and temperature [122]. 

**Figure 5:**
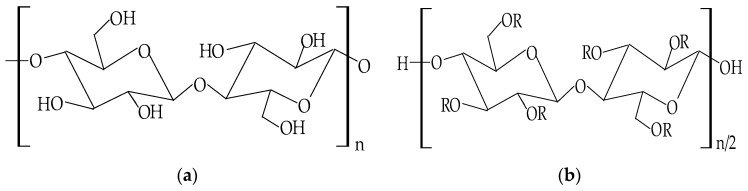
Chemical structures of: (**a**) cellulose; (**b**) cellulose derivatives.

To improve the solubility problems of cellulose and to extend its applications, the chemical structure of this polymer can suffer several changes to obtain the cellulose derivatives, which have suitable physicochemical properties to be used in many fields, especially in the pharmaceutical and biomedical industry [123]. The modifications in the cellulose molecule can be chemical, physical, or biological [114], but the most used and significant of the three is the chemical modification. Targeted by this method are the hydroxyl groups, which suffer an esterification or an etherification reaction [124]. Therefore, the cellulose derivatives can be classified in two major classes: cellulose esters derivatives and cellulose ethers derivatives, which have particular mechanical and physicochemical characteristics [125]. The chemical structures of cellulose and cellulose derivatives are presented in Figure 5.

Cellulose ethers derivatives are characterized by high molecular weight and the greatest applicability in the pharmaceutical domain of all these derivatives are: sodium carboxymethylcellulose (NaCMC), hydroxypropylmethylcellulose (HPMC), methylcellulose (MC), hydroxyethylcellulose (HEC), ethylcellulose (EC), hydroxypropylcellulose (HPC), hydroxyethylmethylcellulose (HEMC) and benzylcellulose (BC) [126,127]. The cellulose ethers are illustrated in Table 1.

Cellulose esters derivatives are extensively used in the pharmaceutical industry as enteric coated drug delivery devices, and they also have excellent properties to form films. There are two categories of cellulose esters: organic and inorganic, but the most common in the pharmaceutical practice are organic esters [129]. Among them are cellulose acetate (CA), cellulose acetate butyrate (CAB), cellulose acetate phthalate (CAP), cellulose acetate trimelitate (CAT), hydroxypropylmethylcellulose phthalate (HPMCP), and hydroxypropylmethylcellulose acetate succinate (HPMCAS). With fewer applications in the pharmaceutical industry are inorganic esters, such as cellulose nitrate (CN) and cellulose sulphate (CS) [130]. The cellulose esters are illustrated in Table 2.

Another classification of cellulose derivatives depends on the water solubility of these polymers; thus, there are described in water-soluble cellulose derivatives and water-insoluble cellulose derivatives. In the first category are included the majority of cellulose ethers (methylcellulose (MC), sodium carboxymethylcellulose (NaCMC), hydroxyethylcellulose (HEC), hydroxypropylcellulose (HPC), hydroxyethylmethylcellulose (HEMC) and hydroxypropylmethylcellulose (HPMC)) [131], while the other cellulose ethers (ethylcellulose and benzylcellulose) and cellulose esters are included in the category of water-insoluble cellulose derivatives. Between the two categories, water-soluble cellulose derivatives are the most used biopolymers in the pharmaceutical and biomedical industry [115,132], because they present several favorable features, such as solubility, surface activity, viscosity in solution, similar properties to thermoplastic film, and proper stability to oxidative and hydrolytic reactions, heat and biodegeneration [130,133]. 

**Figure 6:**
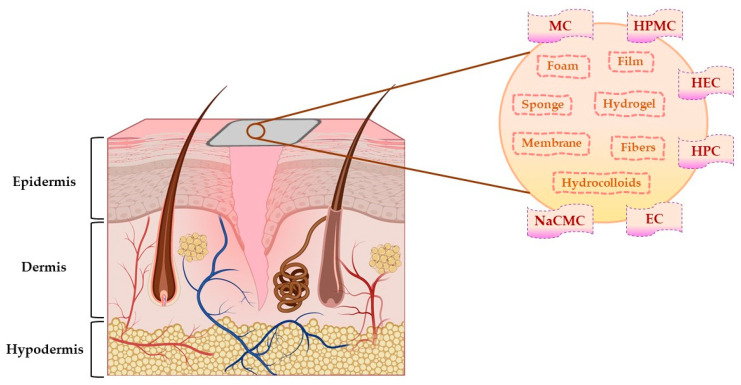
The representation of cellulose derivatives-based wound dressing on an open wound. This illustration has been created with BioRender.com, Inkscape, and PowerPoint.

Due to the general properties of wound dressings presented in Section 4.1, but also the particular properties, such as hydrophilicity, mechanical toughness, pH stability, and rheological characteristics, cellulose and cellulose derivatives have multiple applications in many fields [134]. Areas of the applicability of all these biopolymers involve: biomedical and pharmaceutical industries, where they can act as drug-delivery devices, wound dressings, muco- and bioadhesive drugs, excipients for drug formulations, and support for tissue engineering [29]; also, they can be used for cosmetic and hygienic products, in the textile area, in the food industry and agriculture [128,135]. The representation of cellulose derivatives-based wound dressing on an open wound is illustrated in Figure 6.

Cellulose ethers derivatives (Table 1) are the most used biopolymers for tailoring of new wound dressings, compared to cellulose esters derivatives. Therefore, we will further describe them, and we will present their main different types of wound dressings for an optimal wound management, from gels to foams, as we summarized in Section 4.2.

### 5.2. Sodium Carboxymethylcellulose-Based Wound Dressings

**Figure 7:**
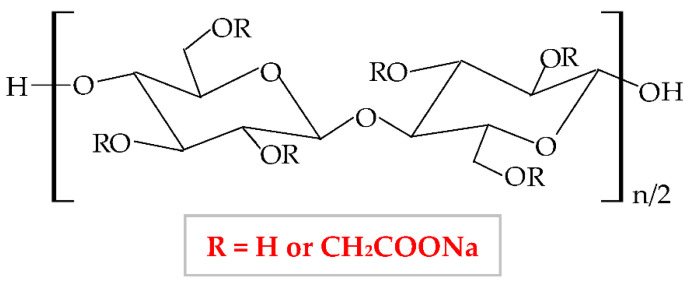
Chemical structure of sodium carboxymethylcellulose (NaCMC).

Carboxymethylcellulose (CMC), also known as carmellose, is a semi-synthetic and hydrophilic polymer, a water-soluble cellulose ether derivative, and one of the polymers with the lowest price [136]. Sodium carboxymethylcellulose (NaCMC) is the sodium salt of CMC, an anionic polymer, with a great solubility in water [137]. NaCMC was the first compound from the group of cellulose derivatives; therefore, all the researchers’ attention was focused on it because, compared to other cellulose derivatives, NaCMC can be synthesized through simple methods with low-cost materials [138]. It results from the etherification reaction of the cellulose with sodium monochloroacetate in an alkaline solution (NaOH) [139]. In the cellulose molecule, three hydroxyl groups (from 2, 3, and 6 positions) are substituted by carboxymethyl groups [140], resulting in different values of substitution degree from 0.4 to 1.5 and different molecular weights of NaCMC, varying from 90,000 to 2,000,000 g/mol [51]. The optimal substitution degree to be used in the pharmaceutical industry is from 0.60 to 1.00 [139]. The chemical structure of NaCMC is shown in Figure 7.

The NaCMC network illustrates a thixotropic behavior to generate 3D structures through intermolecular attraction. Its thixotropy is influenced by concentration and degree of substitution [141]. NaCMC presents excellent physicochemical and mechanical properties [142], optimal biocompatibility and biodegradability, proper capacity to absorb the water and to swell, high gelation behavior, non-toxicity, and low-immunogenicity [143]. It is the most used cellulose derivative in the pharmaceutical industry, mainly for the development of new wound dressings because it has the capacity to absorb heavy exudates [144,145], to ensure excellent moisture at the lesion site, and to avoid skin tissues water loss and tissues necrosis. Moreover, an optimal local humidity can impede dehydration, facilitate the synergy between target cells and growth factors, promote angiogenesis advancement, the mitigation of the ache, and the disruption of the fibrin network [146]. NaCMC is also used as a drug-delivery device and excipient for drug formulations (used as an emulsifier, thickener, stabilizer, and film-maker) [147]. Besides its applicability in the pharmaceutical area, this biopolymer possesses different usefulness in the food (E466 food additive) industry [148], in paper, textile and cosmetics domains [51,149], for tissue culture and dental medicine field [150]. 

NaCMC can be combined with other polymers to enhance its properties and to develop its applicability. Thus, it is more advantageous to blend two or more polymers for the development of a new material comparative to the chemical industrial development of that material. Moreover, the new material obtained by mixing other well-known polymers presents all the properties or is more favorable than the component polymers [75]. Furthermore, the blend of polymers can be realized to compensate for their drawbacks. Hence, Liu et al., combined NaCMC with HEC by electrostatic complexing and obtained a sponge and a membrane with a porous network, enhanced viscoelastic properties, and high swelling behavior [151]. Hu et al., mixed NaCMC with PVA and quaternized chitosan and designed a new composite with enhanced flexibility, water absorption rate, mechanical strength, swelling ratio, and humidity permeability [152]. A novel NaCMC/PVA-based composite was formulated, with higher properties than two polymers: improved swelling capacity, elasticity, water solubility, porosity, water vapor transmission rate, bioavailability, and biodegradability for the tissue repair process; this formulation also presented an extension of its applicability, such as agriculture, biomedical field as drug delivery systems and food packaging [153,154]. NaCMC was blended with PEG through a photo-click reaction based on thiol-norbornene. It formed a pH-sensitive hydrogel with an augmented swelling ratio [150]. Zhang et al., designed a novel hydrogel based on NaCMC and sodium alginate. In a ratio of 1:4, the hydrogel exhibited high biocompatibility, mechanical characteristics, degradation rate, and local humidity [155]. Shin et al., blended NaCMC with PVA and PEG 400 through cyclic freezing/thawing method and obtained a hydrogel with improved properties: the swelling rate, the compressive strength, and cytocompatibility [156]. NaCMC can also be blended with diverse biopolymers to develop a new potential wound dressing with better properties to accelerate the wound healing process. All these combinations are illustrated in Table 3.

### 5.3. Hydroxypropylmethylcellulose-Based Wound Dressings

**Figure 8:**
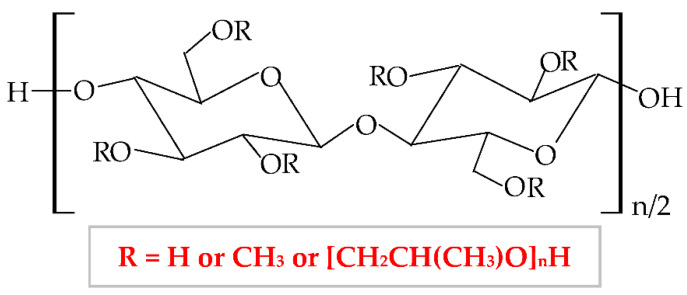
Chemical structure of hydroxypropylmethylcellulose (HPMC).

Hydroxypropylmethylcellulose (HPMC), hypromellose [187], is a semi-synthetic hydrophilic polymer, a nonionic cellulose ether derivative [188], with higher stability at a lower pH. In terms of physical properties, HPMC is a white, fibrous, or granular powder, whose particles are not cohesive, and it does not have a taste and odor [189]. This biopolymer results from hydroxyl groups substitution from cellulose molecule with methyl and hydroxypropyl groups. The chemical structure of HPMC is illustrated in Figure 8.

Therefore, HPMC presents many degrees of substitution, that give to this biopolymer different molecular weight and physicochemical features (rheological properties and crystalline nature) [190,191]. The hydrophilic or hydrophobic nature is related to the values of the degree of substitution (DS) and the molar substitution (MS). Thus, the HPMC molecule with decreased values of DS and MS is more hydrophilic and the HPMC molecule with increased values of DS and MS is more hydrophobic [192]. Following this chemical substitution, HPMC gets both polar (hydroxypropyl) and non-polar (methyl) character; consequently, it can form hydrophobic, intermolecular, and intramolecular linkages with many other materials [190]. The non-ionic character leads to a limited adhesive capacity [193]. At high temperature, the biopolymer can suffer a thermoreversible phase transition from sol to gel, with a temperature of gelation over 60 °C, superior to the temperature of the body (37 °C) [194]. HPMC-based hydrogels are temperature-responsive [195]. 

According to United States Pharmacopeia (USP), there are four distinct forms of HPMC, which are categorized by the content of methoxy, respectively hydroxypropoxy groups in: HPMC 1828, HPMC 2208, HPMC 2906, and HPMC 2910 [196]. This biopolymer has been approved as a food additive, E464 [197], by the American Institute, Food and Drug Administration (FDA), by the European Institution, European Parliament, and Council Directive, and by the Joint Expert Committee on Food Additives [198]. 

HPMC has a proper solubility in water, and it is one of the most used cellulose derivatives in many industries. It is widely used in the biotechnological field (construction, food, cosmetics, biomedical, and pharmaceutical industry), due to its excellent characteristics, such as biocompatibility, biodegradability, superior stability, large availability, excellent swelling, high surface activity, and mechanical properties [199], remarkable ability to form films and poor toxicity [200]. Regarding the applicability of HPMC in biomedical and pharmaceutical domains, it is used as a drug-delivery device, with a large practice for wound dressings development and it can also have remarkable applicability in tissue engineering [201]. HPMC can also be used as an excipient because it possesses proper abilities of emulsification, stabilization, suspension, and thickening [202,203]. 

HPMC can be combined with other polymers to enhance its properties and to develop its applicability [204]. To improve the physicochemical properties of a new composite, HPMC has been blended with several natural, semi-synthetic, or synthetic polymers [205]. In this way, to improve the thermal stability, HPMC has been blended with collagen [206,207], gelatin [204], chitosan [208], chitosan, and xanthan gum [209]; to improve the mechanical properties (tensile strength and ultimate elongation), HPMC has been mixed with chitosan [210], collagen [207], poloxamer 407 [211], silk fibroin [212], PVA and PVP [213], chitosan and xanthan gum [209]; to increase the swelling rate, HPMC has been combined with methylcellulose [214], κ-carrageenan [215], chitosan and hyaluronic acid [216], chitosan and xanthan gum [209]. HPMC can also be blended with diverse biopolymers and multiple bioactive agents (plants extracts, organic or inorganic substances, and chemical drugs) to develop new potential wound dressing to accelerate the wound-healing process. All these studies are summarized in Table 4.

### 5.4. Methylcellulose-Based Wound Dressings

**Figure 9:**
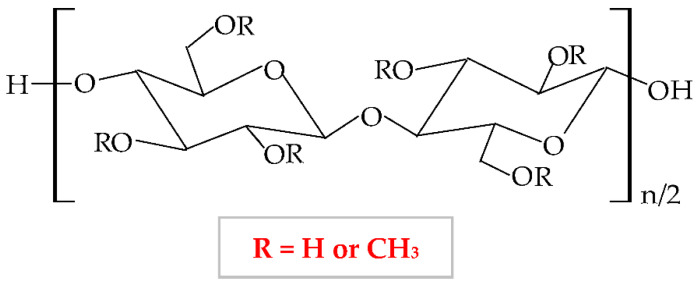
Chemical structure of methylcellulose (MC).

Methylcellulose (MC) is a semi-synthetic and non-ionic polymer, a cellulose ether derivative with high solubility in water, which is influenced by temperature [247]. It forms through the etherification of cellulose molecule with methyl chloride or dimethyl sulfate in basic solution [27] when the hydroxyl groups from the mother molecule are substituted with methyl groups, which leads to a diminishing of crystallinity [248]. The chemical structure of MC is presented in Figure 9. 

At a variation of temperature, MC has a thermo-sensitive behavior with a reversible sol-gel transition in an aqueous solution [249]. At a lower temperature than lower critical solution temperature, it realizes the hydration of the MC network in solution, with the formation of hydrogen bonds. At a higher temperature than lower critical solution temperature, the MC aqueous solution takes in the heat, with the disintegration of hydrogen bonds [195]. Thus, MC presents increased viscosity at higher temperatures, and at lower temperatures it exhibits a reduced viscosity [250]. 

The degree of substitution for commercial MC varies from 1.7 to 2.2 when it results in a semiflexible biopolymer because the inter-and intra- hydrogen bonds from cellulose molecule break off [251]. There are many substances, which influence the gelation behavior of MC, such as inorganics salts, ethanol, propylene glycol, polyethylene glycol 400, sucrose, glycerin, sorbitol, and different surfactants (sodium dodecyl sulfate and cetyltrimethylammonium bromide) [252]. MC is extensively used in biomedical, pharmaceutical, cosmetic, and food industries as a thickening, binding, and film-forming agent because it possesses excellent biocompatibility, biodegradability, and reduced toxicity [253,254,255]. 

To improve the characteristics of MC, it can be blended with other polymers in different ratios to enhance the physicochemical, morphological, and structural properties of both polymers and of the resulting composite [255]. Abu et al., illustrated that a higher concentration of MC led to increased hydrophilicity and porosity of the MC-chitosan scaffold due to the hydroxyl groups from the MC molecule, which can attract water molecules. The higher wettability has been described by the suitable results of the water uptake capacity [256]. Another combination of MC and chitosan was studied by Tan et al., They illustrated that an augmented concentration of MC led to improved tensile strength, moisture content, whitish index, and elongation at break [257]. El-Naggar et al., mixed MC with PVA and doxycycline hyclate (drug model) to develop a new drug delivery device, which showed a proper swelling capacity and a high drug release at basic medium [253]. The combination between MC and poly(acrylic acid) presented optimal mechanical properties and thermal stability [258]. The novel composite resulting by blending MC and tragacanth gum exhibited a higher capacity to form a gel and adequate mechanical and rheological properties [259]. MC can also be blended with diverse biopolymers and multiple bioactive agents (plants extracts, organic or inorganic substances, and chemical drugs) to develop a new potential wound dressing to accelerate the wound-healing process. All these mixtures are presented in Table 5.

### 5.5. Hydroxyethylcellulose-Based Wound Dressings

**Figure 10:**
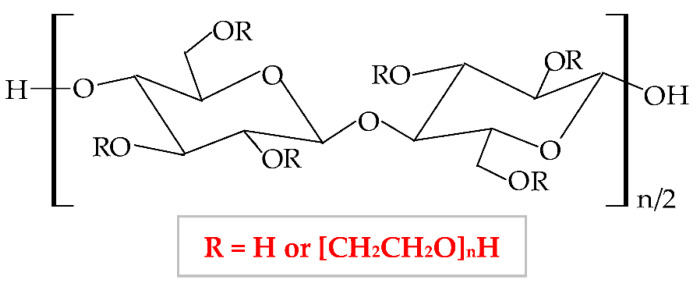
Chemical structure of hydroxyethylcellulose (HEC).

Hydroxyethylcellulose (HEC) is a semi-synthetic, nonionic, and inert polymer, a water-soluble cellulose ether derivative [289]. It forms through etherification of alkaline cellulose with chlorohydrin or ethylene oxide, when hydroxyl groups from cellulose molecule are substituted with hydroxyethyl groups [290]. The chemical structure of HEC is illustrated in Figure 10.

It has a low price, without taste and smell, with no color to light yellowish [291]; presents optimal stability at pH values between 2 and 12 [292]. HEC exhibits a proper capacity to scavenge free radicals and to form hydrogen and electrostatic bonds [293]. HEC is regarded as a hydrogel-like material, with two important characteristics: liquid-like and solid-like. Due to its polysaccharide structure, this hydrophilic biopolymer exhibits a high capacity to absorb and hold a large quantity of water or wound exudates. The elastic strength of its structure leads to an expansion of the molecule dimensions, without the modification of the structural stability and the gel form [294]. HEC possesses excellent physicochemical properties: rheological, hydrodynamic, and thermodynamic [295]. HEC also presents adequate biocompatibility, biodegradability, insignificant toxicity, immunogenicity, and cementing properties [296]. Due to its nonionic behavior, HEC exhibits the ability to coexist with a large field of other polymers, which have an appropriate solubility in water, salts, or surfactants. Therefore, HEC presents optimal toughness in a dielectric solution with a large concentration [297]. This biopolymer presents the largest commercial availability from all cellulose derivatives [298]; therefore, HEC is a noticeable biopolymer, which can be used successfully as an emulsifier, film-coating, stabilizer, suspender, and thickener agent in biomedical, pharmaceutical (wound dressing development) [299], cosmetic, food, adhesive, and textile industries [291,300,301]. The most predictive method for hydrogels synthesis is the crosslinking of free radicals generated by irradiation (electron beam and gamma-radiation) [302].

To enhance its properties, HEC can be blended with other polymers. For example, Zia et al., mixed HEC with poly(lactic acid) and polyurethane. They obtained a new composite with higher thermal stability and mechanical (tensile strength and elongation) properties compared to other polymers [303,304]. Moreover, HEC has been blended with polyvinyl alcohol (PVA), resulting in suitable electrical conductibility, viscoelasticity, stretchability, and thermosensitivity [305]. Guo et al., combined HEC with poly(caprolactone) by trimethylsilyl group technology and the result was the formation of a new copolymer with enhanced thermal properties [306]. HEC was also blended with chitosan to obtain a copolymer with improved physicochemical and mechanical characteristics [307], with gelatin to obtain a superparamagnetic composite [308], with sodium alginate to form a copolymer with enhanced swelling efficacy and drug delivery profile. HEC can also be combined with diverse biopolymers to formulate novel wound dressing, which can stimulate the wound healing process and restore the damaged skin. The main combinations are summarized in Table 6.

### 5.6. Ethylcellulose-Based Wound Dressings

**Figure 11:**
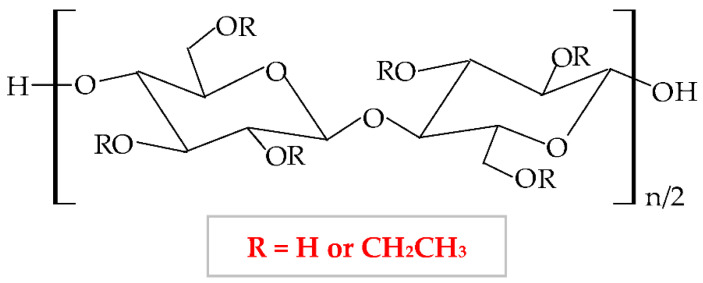
Chemical structure of ethylcellulose (EC).

Ethylcellulose (EC) is a nonionic semi-synthetic polymer, a cellulose ether derivative insoluble in water [318]. It forms through the etherification of alkali cellulose with ethyl chloride when the hydroxyl groups from cellulose molecule are substituted with ethyl groups [27]. The chemical structure of MC is presented in Figure 11.

This biopolymer presents numerous advantageous characteristics, such as mechanical properties, biodegradability, flexibility, low toxicity, hydrophobicity, gelling capacity [319], light, moisture, oxygen resistance, thermoplasticity [320], and low price, which make EC an excellent material for use in different industries (pharmaceutical, cosmetic and food) [321]. Moreover, this biopolymer has several particular features in addition to the other cellulose derivatives: high film-forming capacity, suitable chemical strength, and optimal mechanical properties [322]. EC represents the most extensively analyzed biopolymer due to its capacity to form film for coating solid pharmaceutical forms (tablets, microcapsules, and microspheres) and formulation of new topical forms [323]. EC is a promising material to be used for encapsulation due to its optimal optical transparency, processing temperature, and electronic insulation [324]. It also presents a good capacity to bind, preserve and dissolve [325], and possesses a proper control of drug delivery [326]. Films based on EC are brittle because of the stiffness of hydrogen linkages from its molecule. This biopolymer has high stability to chemical substances and can be associated with different plasticizers to design heavy and impermeable films [327].

EC can be mixed with various polymers to enhance the physicochemical and mechanical properties and thus, its applicability. To develop a novel drug-delivery device, Li et al., blended EC by electrospinning method with poly(di(ethylene glycol) methyl ether methacrylate), a thermosensitive polymer. The new formulation showed normal morphology, a large porosity, and an increased wettability at a higher temperature, which led to more hydrophobic behavior, causing an extended release of the drug [328]. EC was mixed with poly (ethylene-co-vinyl acetate) and resulted in a new composite with higher mechanical properties [329]. Chen et al., mixed EC and poly(β-hydroxybutyrate) when EC acted as a thickening agent because it increased the viscosity of the new composite. In a concentration of 1%, EC augmented the tensile strength [330]. Li et al., blended EC with konjac glucomannan to formulate a novel composite with higher mechanical properties, moisture resistance, permeability of oxygen, and stability at a high temperature [331]. EC was also associated with another cellulose derivative, HPC, and obtained a scaffold with enhanced mechanical properties and 3D printing capacity [332]. 

EC can also be combined with other polymers to develop new wound dressings, with enhanced physicochemical and mechanical properties that can accelerate the wound-healing process. Principal blends are presented in Table 7.

### 5.7. Hydroxypropylcellulose-Based Wound Dressings

**Figure 12:**
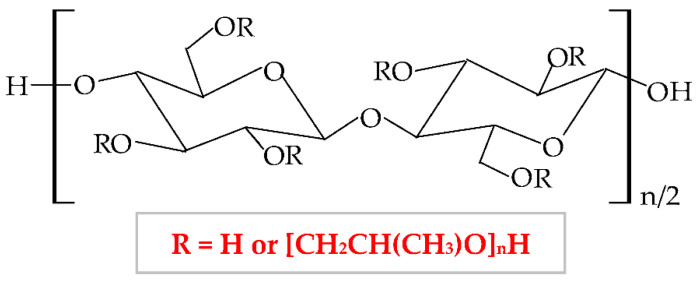
Chemical structure of hydroxypropylcellulose (HPC).

Hydroxypropyl cellulose (HPC) is a semi-synthetic hydrophilic polymer, a cellulose ether derivative, with proper solubility in water and organic solvents [339]. Its solubility depends on the degree of substitution. At values smaller than 12%, HPC is water-soluble and at values higher than 12%, HPC is ethanol-soluble [340]. This biopolymer results from the etherification reaction of alkali cellulose with 1,2-propylene oxide. Thus, the 2,3,6-hydroxyl groups from the cellulose molecule are replaced with hydroxypropyl groups [27]. The chemical structure of HPC is presented in Figure 12.

It has numerous advantageous properties, such as amphiphilicity, low price, electrical neutrality, biocompatibility, biodegradability, non-toxicity, high power of swelling the wounds exudate [341,342], adequate chemical strength, and film-forming efficiency [236]. At a high temperature and in a concentrated aqueous solution, HPC generates a cholesteric liquid crystalline network, depending on its concentration [343]. HPC exhibits a thermoplastic behavior and develops temperature-responsive hydrogels [195,344]. Regarding the HPC-based films, these are defined by high flexibility, good impermeability for oil and fat, and a low value of T_g_ (glass transition temperature) at excessive humidity. The LCST (lower critical solution temperature) water value is about 41 °C. At a slightly higher temperature than LCST, HPC presents a phase change because the water solution of this biopolymer generates metastable nanosphere aggregates [345]. Moreover, the solubility of HPC is influenced by LCST values. At a lower temperature than LCST, HPC dissolves easily in water and at a higher temperature than LCST, HPC does not dissolve [346]. Thus, this cellulose derivative is an optimal material to be used in biomedical and pharmaceutical fields as a binding, disintegrating, emulsifying, thickening, filler, and coating agent [347,348] and in the construction domain [349]. It can also be used in the food industry because the United States Food and Drug Administration (FDA) authorized HPC as a safe food additive [350].

HPC can be blended with other polymers to improve the physicochemical and mechanical properties and thus, to extend its applicability. For instance, Veerapur et al., combined HPC and chitosan, and the new formulated composite presented higher hydrophilicity, swelling capacity, and permeation rate [351]. By mixing HPC with cellulose acetate phthalate resulted a composite with higher properties than compounds: increased pseudoplasticity and viscoelastic behavior [352]. Gan et al., prepared a high-performance hydrogel with enhanced tensile strength, toughness, biocompatibility, wear resistance, and low friction coefficient from HPC, sodium alginate, and poly(vinyl alcohol); these excellent characteristics extend the area of use to biosensors and nerve replacement [353]. Lu et al., blended HPC with poly(vinyl alcohol) to obtain a new scaffold with augmented toughness, elasticity, conductivity, and mechanical strength that is a promising material for the development of biosensors and interaction between humans and machines [354]. HPC can also be mixed with other polymers to develop novel wound dressing, with higher physicochemical traits that can restore the impaired skin tissue. The main combinations are summarized in Table 8.

### 5.8. Combinations of Cellulose Derivatives-Based Wound Dressings

One or more cellulose derivatives may combine with other cellulose derivatives to formulate novel wound dressings, with enhanced properties that can accelerate the wound-healing process and alleviate the pain, inflammation, and stress caused by damaged skin tissue. Moreover, they can be combined to counteract their drawbacks [75]. The main combinations are summarized in Table 9.

## 6. Conclusions and Future Perspectives

This review has focused on different types of wound dressing (gel, hydrogel, sponge, hydrocolloid, film, membrane, foam, and nanofibers) based on cellulose derivatives as biopolymeric scaffolds, and various bioactive agents, from plant extracts to chemical drugs. We have considered the cellulose ethers derivatives (NaCMC, HPMC, MC, HEC, EC, and HPC). It has been illustrated that cellulose derivatives can manifest a therapeutic effect on wound healing, alone or in combination with other natural, semi-synthetic, and synthetic polymers. The major advantage of mixing two or more biopolymers is, besides the beneficial action on damaged tissue, the improvement of physicochemical properties of the novel dressing. Cellulose derivatives have a particular chemical structure, obtained by etherification of hydroxyl groups from cellulose molecule with different alkyl groups, the consequence being the improvement of water solubility. Therefore, these biopolymers can be successfully used as a base for diverse formulations, due to their high gelation properties. Cellulose derivatives exhibit an efficient capacity to absorb the exudates on the site of the lesion, retain them, and swell. Consequently, the newly formulated wound dressings show an excellent ability to maintain relevant moisture on the wound bed and allow gas exchanges with the environment. Due to their high biocompatibility, biodegradability, physicochemical properties, eco-friendliness, and low cost, cellulose derivatives are promising materials for biomedical and pharmaceutical domains (electrochemical biosensors for medical diagnosis, bone tissue engineering, hemodialysis, drug delivery and 3D printing), for oilfields, carbon capture and the food industry.

## Figures and Tables

**Table 1:** Main cellulose ether derivatives according to R groups [128].

Cellulose Ethers	R Groups
Methylcellulose	H, CH_3_
Ethylcellulose	H, CH_2_CH_3_
Benzylcellulose	H, C_6_H_5_CH_2_
Sodium carboxymethylcellulose	H, CH_2_COONa
Hydroxyethylcellulose	H, [CH_2_CH_2_O]_n_H
Hydroxypropylcellulose	H, [CH_2_CH(CH3)O]_n_H
Hydroxyethylmethylcellulose	H, CH_3_, [CH2CH2O]_n_H
Hydroxypropylmethylcellulose	H, CH_3_, [CH_2_CH(CH_3_)O]_n_H

**Table 2:** Main cellulose ester derivatives according to R groups [128].

Cellulose Esters	R Groups
Acetate	H, I
Acetate trimelliate	H, I, II
Acetate phthalate	I, III
Hydroxypropylmthylphthalate	H, CH_3_, CH_2_CH(OH)CH_3_, III, IV
Hydroxypropylmthylphthalate acetate succinate	H, CH_3_, CH_2_CH(OH)CH_3_, III, V

**Table 3:** Recent studies on the use of sodium carboxymethylcellulose as a wound dressing.

Biopolymer/-s	Active Pharmaceutical Ingredient (Natural or Synthetic Substances)	Type of Wound Dressing	Main Findings	References
NaCMC	-	Gel	Anticoagulant activity at second-degree burn injuries; this action was influenced by concentration, substitution degree, and molecular weight of the biopolymer.	[157]
		Hydrocolloid	Appropriate management of humidity, maintained self-adhesiveness, and increase of the surface energy.	[85]
		Film	Enhanced tensile strength, absorption capacity, and decreased percent erosion.	[147]
		Foam	Suitable material for wound dressing, because it can maintain optimal local moisture.	[158]
	Merremia mammosa	Gel	Good gel properties, non-toxicity, speeding the wound healing process in diabetic ulcer (increases collagen synthesis and improves angiogenesis).	[159]
	Chestnut honey	Hydrogel	Enhanced moisture retention, non-adherence, and excellent antibacterial action to treat diabetic ulcer injuries.	[160]
	Reduced graphene oxide	Hydrogel	The inhibition of bacteria biofilm from infected wounds.	[161]
	Neomycin trisulphate, sulphacetamide sodium, and silver nitrate	Hydrogel	Proper elasticity, sponginess, homogenous texture, suitable smell, high drugs delivery, and antimicrobial effect.	[162]
	Tetracycline–UiO-66	Hydrogel	Increased mechanical properties, antibiotic delivery, and good antibacterial activity.	[163]
	ZnO-MCM-41 and tetracycline	Hydrogel	Improved tensile strength, permeability for gases, and swelling ratio; optimal antibacterial effect.	[164]
	Lidocaine hydrochloride	Film	Optimal physicochemical and mechanical properties; high drug release (90%) in the first 15 min.	[165]
	AgNPs	Membrane	Good morphology and superior antibacterial activity.	[166]
NaCMC/Chitosan	-	Sponge	High porosity, air permeability, and proper antibacterial effect; in the presence of the cyanine dye, the sponge indicated the pH of the wound site.	[167]
NaCMC/Pullulan	-	Hydrogel	An important decrease of the hydrogel adherence at the postoperative lesions.	[168]
NaCMC/Sericin	-	Hydrogel	Increased mechanical stability and the strength to hydrolysis and enzymatic degeneration; the molecular weight of CMC influenced the sericin release.	[169]
		Film	Higher mechanical properties, hydrophilicity, swelling power, stability, compatibility with cells and blood, and stimulation of cell proliferation.	[170]
NaCMC/ε-Polylysine	-	Hydrogel	Optimal rheological properties, degradability, compression modulus, and high antibacterial activity.	[171]
NaCMC/Pectin/Cellulose	-	Film	NaCMC and cellulose enhanced the mechanical properties of pectin; the new composite accelerated the tissue repair through re-epithelialization and collagen deposition.	[172]
NaCMC/Gelatin/Pectin	-	Film	Excellent capacity to absorb biological fluid, elongation at break, mechanical properties, and water vapor transmission rate; ideal to cure infected moist injuries.	[173]
NaCMC/Chitosan/Sodium alginate	-	Hydrogel	Optimal water vapor permeability, anti-adhesion capacity to treat second-degree burn injuries; reduced the TNF-α and IL-6 levels.	[174]
NaCMC/Gelatin/PEG	-	Hydrogel	CMC increased the mechanical properties, enzymatic stability, and sanguineous compatibility; the new hydrogel had 3D and porous structure, absorbed the exudates, and maintained proper moisture.	[175]
NaCMC/PEG	-	Hydrogel/Film	Superabsorbent hydrogel with various swelling degrees from 100% to 5000%, appropriate physicochemical and morphological properties to regenerate chronic wounds.	[45]
NaCMC/PE/PP	-	Fibers	A serious increase of wet strength; future perspective as a material for the treatment of wounds with mild exudate.	[176]
NaCMC/Collagen	Mefenamic acid	Hydrogel	Good ability to swell, optimal morphological structure and excellent release model to treat burns.	[177]
	AgNPs	Membrane	Suitable antimicrobial action against pathogens from infected burn wounds.	[178]
NaCMC/Gelatin	Ciprofloxacin	Film	In a ratio of 1:1 and 1:2, the film showed good thickness, sorption capacity, blending endurance, and proper antibacterial effect.	[16]
NaCMC/Keratin	Clindamycin	Sponge	The keratin enhanced the water stability and the water vapor transmission rate; the sponge impeded the bacterial multiplication.	[2]
NaCMC/PEO	*Achillea millefolium*, *Calendula officinalis*, *Matricaria chamomilla*, *Echinacea purpurea* and *Hypericum perforatum*	Fibers	Antibacterial and antioxidant activity for acute injuries treatment.	[179]
	Lidocaine	Nanofibers	Similar characteristics as extracellular matrix; ~50% of lidocaine has been delivered in the first 10 min, relieving the pain quickly.	[180]
	AgNPs	Nanofibers	Smooth surface and limited size distribution; excellent antimicrobial effect and photoluminescent act, used as anti-adhesion composite and wound dressing.	[181]
NaCMC/PVA	Propolis	Hydrogel	High swelling power and antimicrobial effect for second-degree burn injuries treatment.	[182]
	ZnO and heparin	Hydrogel	Favorable mechanical properties, water vapor transmission rate, swelling ratio, and excellent antibacterial effect.	[183]
NaCMC/Soluplus^®^	18β-glycyrrhetinic acid (licorice)	Hydrogel	Soluplus^®^ augmented the solubility in water of the bioactive agent; at the lesion site, and in situ hydrogel formed, which had high swelling power and was easy to eliminate by washing.	[184]
NaCMC/Sodium alginate/PET/Viscose	Diclofenac sodium	Membrane	Optimal drug release and efficient anti-inflammatory activity.	[185]
NaCMC/Sodium alginate	Diclofenac	Film	Homogeneous film with proper water vapor transmission rate; faster alleviation of pain.	[186]

Abbreviations: AgNPs—Silver (Ag) nanoparticles, MCM-41—Mesoporous silica, PE—Polyethylene, PEG—Polyethylene glycol, PEO—Polyethylene oxide, PET—Polyethylene terephthalate, PP—polypropylene, PVA—Polyvinyl alcohol, Soluplus^®^—Polyvinyl caprolactam-polyvinyl acetate-polyethylene glycol graft copolymer (PCL-PVAc-PEG), UiO-66—The University of Oslo, a metal-organic framework based on Zr, ZnO—Zinc oxide.

**Table 4:** Recent studies on the use of hydroxypropylmethylcellulose as a wound dressing.

Biopolymer/-s	Active Pharmaceutical Ingredient (Natural or Synthetic Substances)	Type of Wound Dressing	Main Findings	References
HPMC	*Lawsonia inermis* (henna) and *Matricaria chamomilla*	Gel	Adequate stability, extrudability, viscosity, homogeneity, good herbal extract release, and antibacterial effect for burn infection cure.	[217]
	Liposomal farnesol	Gel	The combination of HPMC:farnesol in a ratio of 1:2 and 2:1 had an excellent effect on tissue regeneration at third-degree burns.	[218]
	Bacteriophage	Gel	The gel containing 3% HPMC presented good stability at 37 °C and had an effective antibacterial action against *Klebsiella pneumoniae* in wound infection treatment.	[219]
	CuNPs-licorice and phenytoin	Gel	Effect on acute lesions by the suppression of the inflammatory JAK3 and the synthesis of the procollagen type I.	[220]
	Honey and Aloe vera	Hydrogel	At 3% concentration, the hydrogel presented an adequate viscosity to be applied on the burn lesions and showed a proper antibacterial activity on infection with *Klebsiella pneumoniae*.	[197]
	Cefotaxime sodium	Hydrogel	The hydrogel containing 3% HPMC 400 exhibited a high spreadability and released all the drug content after 4 h.	[221]
	Haruan/fusidic acid	Film	Films with 1% plasticizers, respectively 2% plasticizers presented a suitable elongation at break and water vapor permeability.	[222]
	Epigallocatechin-3-gallate	Film	Enhanced tensile strength and water vapor barrier trait.	[223]
	Cooper nanoparticles	Film	Appropriate antibacterial activity.	[190]
HPMC/Silk fibroin	-	Nanofibers	The mixture of HPMC:silk fibroin in a ratio of 7:1 exhibited suitable biocompatibility, mechanical properties, hydrophilicity, and porosity for skin tissue engineering.	[201]
HPMC/Collagen/Polyurethane	-	Hydrogel	Increased proteolytic and thermal degradation.	[224]
HPMC/Polyacrylate/Tri-isocyanate crosslinked polyurethane	-	Hydrocolloid	The newly designed wound dressing containing 1% cross-linking promoter showed proper humidity (the capacity of the water uptake was 5.1% after 1 h).	[199]
HPMC succinate/Chitosan	Gentamycin sulfate	Film	High mechanical properties and antibacterial activity.	[225]
HPMC/Chitosan	Simvastatin	Gel	High viscosity and bioadhesive strength.	[17]
	Toluidine blue O	Hydrogel	High viscosity, hardness, bioadhesion, and bactericidal effect to alleviate the burn wounds caused by light irradiation.	[226]
	Pioglitazone hydrochloride	Hydrogel	The formulation containing HPMC E5:chitosan (1:2) showed the best physicochemical properties and the highest drug release.	[193]
	Silver sulphadiazine	Film	In a ratio of 1:1, the film showed the best physicochemical properties and the highest drug release; the period of wound healing was of 8 days in comparison with the marketed cream (14 days).	[227]
HPMC K100/Collagen	Curcumin	Nanogel	In vivo studies illustrated a substantial rate of lesion contraction (95.42 ± 12.20%) on the 20th day.	[228]
HPMC/Hydroxyapatite	AgNPs	Hydrogel	High porous 3D structure, with excellent mechanical properties and antibacterial activity; after 16 days, wounds healed considerably (94.5 ± 1.4%).	[229]
HPMC/Hydroxypropyl-β-cyclodextrin	Coumestrol	Hydrogel	50% of wounds healed in a shorter time compared to the commercial product, with a suitable tissue re-epithelialization after 12 days.	[230]
	Gallic acid	Hydrogel	High physicochemical properties and antibacterial effect to prevent wound infection.	[231]
HPMC/PVA/PAA/PVP/PEG	Aloe vera (1%, 6%, 10%)	Nanofibers	The sample with 10 wt% aloe vera showed higher porosity and durability, faster healing of burns, and nonlinear structure.	[200]
HPMC/PVA/PVP-I/PEG	Aloe vera (2%, 4%, 6%)	Fibers	Fibers with 6% aloe vera were thinner without any beading, which led to a higher porosity of the fibers.	[232]
HPMC K15M/Tara gum	Lawsone	Gel	Suitable homogeneity, uniformity, and drug release; in vivo studies indicated total epithelialization of the excision lesion.	[233]
HPMC K15M/Xanthan gum	Nano calcium oxide	Hydrocolloid	Great mechanical strength, superior flexibility, homogeneous thickness, and higher lesion contraction compared to the commercial product.	[234]
HPMC/Xyloglucan	Gentamicin sulfate	Film	The best formulation of these polymers was in a ratio of 50:50 and it presented reliable physicochemical properties, good drug release, and favorable antibacterial effect.	[235]
HPMC/Sodium alginate	Gatifloxacin	Hydrogel	Advantageous physicochemical properties regarding the tensile strength, the swelling capacity, the elongation, and the drug release.	[236]
HPMC/Pluronic^®^ 127	AuNPs	Gel	Good bioavailability, skin permeation, anti-inflammatory, and antibacterial effect, with an excellent drug release of 98.03% after 6 h.	[237]
HPMC/Polyacrylamide	AgNPs	Hydrogel	Superporous hydrogel with high porosity (91.4%), which allows a quicker wound healing, with minimal scar formation.	[238]
HPMC/PEO	Beta-glucan	Nanofibers	At the lesion site, the nanofibers produced a hydrogel in situ.	[239]
HPMC/Poly(lactic acid)	Tetracycline hydrochloride	Nanofibers	High water sorption rate and antimicrobial activity.	[240]
HPMC/Polyurethane	Silver and asiaticoside	Foam	Increased absorption capacity and compressive strength development; adequate antimicrobial activity.	[241]
HPMC/Chondroitin sulfate/Sodium hyaluronate	Silver sulphadiazine	Sponge	Proper elasticity, softness, flexibility, and bioadhesive properties, alongside antibacterial activity.	[46]
HPMC K100M/Gum Odina/Gelatin	Fluconazole and ofloxacin	Sponge	The formulation with gum Odina-HPMC K100M:gelatin (1:1) showed excellent physicochemical properties and antimicrobial activity to cure chronic wounds.	[242]
HPMC/HA/Methyl-β-cyclodextrin	Curcumin	Film	Excellent antimicrobial effect.	[243]
HPMC/Hydroxypropyl-β-cyclodextrin/Chitosan	Caffeic acid	Hydrogel	Drug delivery system with superabsorbent capacity, higher swelling property at pH 7, and good antimicrobial effect to prevent wound infection.	[244]
HPMC/Chitosan/Sodium alginate	Lidocaine chloride and polymyxin B sulphate	Biomembrane	Proper mechanical properties (elasticity, tension, stiffness) and thickness; in vivo: high antimicrobial effect for tissue regeneration.	[245]
HPMC/Polyglycolic acid/Vicryl^®^/Catgut	Ofloxacin	Hydrogel	Proper physicochemical properties, which ensures a high lesion size contraction after 14 days (95%) and large collagen deposition on the 21st day.	[246]

Abbreviations: AgNPs—Silver (Ag) nanoparticles, AuNPs—Gold (Au) nanoparticles, CuNPs—Copper (Cu) nanoparticles.

**Table 5:** Recent studies on the use of methylcellulose as a wound dressing.

Biopolymer/-s	Active Pharmaceutical Ingredient (Natural or Synthetic Substances)	Type of Wound Dressing	Main Findings	References
MC	Zinc oxide and Silver	Ointment	MC 3% ointment showed an elastic behavior and thixotropy, which can be used for injured skin with complex relief.	[260]
	*Allium hirtifolium*	Gel	In a ratio of 1:1, the newly developed gel accelerated the open wounds healing through the tissue re-epithelialization.	[261]
	Cryopreserved human culture of fibroblasts and AuNPs	Gel	High capacity to heal the third-degree burns because it restored the composition of type I and III collagen on 21st day of the treatment.	[262]
	Vitamins (C, B_1_, and B_6_)	Hydrogel	Enhanced gelation rate and mechanical strength, with suitable applicability for wound treatment.	[263]
	Gallic acid and doxycycline	Hydrogel	At body temperature, the novel formulation formed an in situ gel and released the bioactive agents to heal the deep tissue injuries.	[252]
	Neomycin trisulphate, sulphacetamide sodium, and Silver nitrate	Hydrogel	New formulation exhibited elasticity, sponginess, homogenous texture, proper smell, and white color.	[162]
	Silver oxide nanoparticles	Hydrogel	Thermo-responsive hydrogel, which led to wound burn regeneration due to its superior antimicrobial effect.	[264]
	Pedilanthus tithymaloides	Film	A concentration of 0.5% of the plant extract accelerated the re-epithelialization of the wounded skin.	[265]
	AgNPSs	Film	Increased water absorption capacity and contact angle value; the temperature influenced the release of AgNPs.	[266]
	Borate bioactive glass and Manuka honey	Foam	The foam exhibited high porosity, better wettability, mechanical properties, and antibacterial effect.	[248]
MC/α-Chitin nanocrystals	-	Hydrogel	The novel nanocomposite presented high mechanical strength and gelation rate, being a promising dressing for tissue engineering.	[267]
MC/Fucoidan	-	Film	The newly designed formulation could be a promising dressing for wounds with a smaller production of exudates because it possessed a lower capacity to swell.	[268]
MC/Nano hyaluronic acid	-	Hydrogel	The mixture of these polymers led to an in situ hydrogel development, which enhanced tissue regeneration.	[269]
MC/Mucin	-	Hydrogel	MC enhanced the mechanical properties of the mucin and formed a thermoresponsive gel, which can be used for different wounds treatment.	[270]
MC/2-Methacryloyloxy ethyl phosphorylcholine	-	Hydrogel	It reduced the postoperative adhesion effect by inhibition of collagen proteins.	[271]
MC/Gelatin/Gellan gum/PVP	-	Film	The blend of all these polymers led to a novel wound dressing with suitable physicochemical properties to restore minor lesions.	[272]
MC/Chitosan	Adenosine and vitamin C	Hydrogel	Thermo-responsive and self-healing ability and a higher release of adenosine.	[273]
	Exosomes	Hydrogel	Suitable mechanical properties, good gelation time, and excellent self-healing for treatment of severe tissue injuries.	[274]
MC/Hyaluronic acid	-	Gel	It was shown that a low molecular weight of hyaluronic acid increased the biocompatibility and the thermogelation of this new formulated composite.	[275]
	AgNPs	Hydrogel	Excellent morphological, swelling, and spectral properties, together with the high antibacterial effect to treat burn wounds in children (99.6%).	[276]
MC/sECM	Stem cells	Hydrogel	At the wound site, the hydrogel had a thermosensitive sol-gel transition, which accelerate the wound healing through neovascularization and re-epithelialization.	[277]
MC/Sericin	Tranilast	Ointment	The combination of the two polymers and the antiallergic drug exhibited excellent results to augment wound healing and to decrease the redness in diabetic rats.	[278]
MC/Silk fibroin	5-Aminosalicylic acid	Hydrogel	The gelation time of the novel composite has been enhanced by adding MC; thus, this hydrogel presents important uses as a wound dressing and drug release system.	[279]
MC/Sodium alginate	Manuka honey, aloe vera, and eucalyptus essential oil	Hydrogel	The formulation presented proper swelling capacity, biocompatibility, and suitable antimicrobial and antibiofilm action.	[280]
	Gallium (Ga^3+^)	Hydrogel	It was illustrated that this novel hydrogel is a promising dressing for wounds infection prevention due to its large antibacterial action (99.99%) and cytocompatibility.	[281]
	Montmorillonite	Film	A high content of montmorillonite led to higher tensile strength and antibacterial activity of the newly designed film.	[282]
MC/Sodium alginate/Poly(*N*-isopropylacrylamide)	Octenisept^®^	Hydrogel	The novel hydrogel had a large viscosity, an expressed shear-thinning nature, high antibacterial action, a homogenous and microporous structure.	[283]
MC/Pluronic F-127	GT/siMMP9	Hydrogel	It was demonstrated that this new dressing had thermosensitive traits by forming in situ and it reduced the MMP-9 level in diabetic chronic injuries.	[284]
MC/Polyacrylamide	*Aloe barbadensis*	Hydrogel	At a 2% concentration of *Aloe barbadensis*, the hydrogel showed suitable thermal stability; it also presented an antibacterial effect to cure chronic cutaneous wounds.	[285]
MC/Poly(ε-caprolactone)	Bioactive glass and Manuka honey	Fibers	The blend of these two polymers led to higher mechanical properties and wettability.	[286]
MC/PVA	Asiaticoside	Film	This newly formulated dressing exhibited significant mechanical properties, flexibility, transparency, and a shorter healing time of skin wounds.	[287]
	-	Nanofibers	Adequate physicochemical features and antibacterial activity to be used as a drug-delivery system for injuries management.	[288]

Abbreviations: AgNPs—Silver nanoparticles, AuNPs—Gold nanoparticles, GT/siMMP9—Glycogen triethylenetetramine/small interfering RNA matrix metalloproteinase 9, PVA—Polyvinyl alcohol, PVP—Polyvinylpyrrolidone, sECM—Soluble extracellular matrix.

**Table 6:** Recent studies on the use of hydroxyethylcellulose as a wound dressing.

Biopolymer/-s	Active Pharmaceutical Ingredient (Natural or Synthetic Substances)	Type of Wound Dressing	Main Findings	References
HEC	-	Hydrogel	Good antibacterial effect; at a concentration between 12.5% and 15% of HEC, the water absorption rate was the highest.	[309]
HEC	Curcuma longa	Hydrogel	Suitable porous network, mechanical and release properties; besides therapeutic effect, the new hydrogel can illustrate the pH of the lesion site.	[310]
	WO_3_	Hydrogel	The hydrogel with 0.04% WO_3_ showed the highest capacity to heal the injuries due to optimal antibacterial and anti-inflammatory action.	[300]
	Mesocellular silica foam	Sponge	Adequate cytocompatibility, antibacterial and hemostatic action to heal full-thickness wounds.	[311]
	Graphene oxide	Film	Improved mechanical and thermal properties.	[312]
	AgNPs	Membrane	Limited degradation rate, excellent porosity, and water absorption value.	[313]
HEC/Collagen	-	Film	High swelling ratio, mechanical and thermal characteristics, degradation, and adequate biocompatibility.	[314]
HEC/Collagen/PVA	-	Nanofibers	Suitable water absorption rate and degradation behavior, a promising material for skin tissue engineering.	[315]
HEC/Carboxymethyl chitosan	-	Hydrogel	High biocompatibility, gelation time, water evaporation rate, and swelling power.	[290]
HEC/Sodium alginate/Hydroxyapatite	-	Membrane	High porous network, improved mechanical strength, and rigidity gradient.	[316]
HEC/Hyaluronic acid	-	Hydrogel	Optimal biocompatibility and blood compatibility, gelation time, water evaporation rate, water retention ability, and swelling power.	[295]
	Isoliquiritigenin	Hydrogel	pH-sensitive hydrogel with suitable adhesion, rheological properties, and antibacterial efficacy.	[317]

Abbreviations: AgNPs—Silver nanoparticles, WO_3_—Tungsten oxide.

**Table 7:** Recent studies on the use of ethylcellulose as a wound dressing.

Biopolymer/-s	Active Pharmaceutical Ingredient (Natural or Synthetic Substances)	Type of Wound Dressing	Main Findings	References
EC/Gum tragacanth	Honey	Nanofibers	The ratio of 85:15 with 20% honey showed the best mechanical, biological, and antibacterial properties to be used as an efficient wound dressing.	[321]
EC/PVP	Naproxen	Nanofibers	The ratio of 4:1 had the fastest release of the drug; thus, it can be used as a dressing to reduce the inflammation and the pain in acute wounds.	[333]
	AgNPs and ciprofloxacin	Nanofibers	Uniform and cylindrical morphology, with over 90% release of ciprofloxacin and a high antibacterial effect.	[334]
EC/Poly(3-hydroxybutyrate)	p-4-hydroxybenzoic acid and ferulic acid	Film	Potent bacteriostatic and bactericidal action to heal wounds infection facilitating the skin restoration.	[335]
EC/Polylactic acid/Collagen	Silver sulfadiazine	Nanofibers	Good mechanical properties, proper antimicrobial effect, and enhanced cell proliferation.	[336]
EC	Ciprofloxacin	Nanofibers	Cylindrical and homogenous aspect; high antibacterial action.	[318]
EC/Zein	Photosensitizer protoporphyrin and vaccarin	Membrane	Adequate flexibility, hygroscopicity, nanonetwork structure, and mechanical properties; in vivo study showed increased angiogenesis of the injured tissue.	[337]
EC/PVA	Luliconazole	Nanosponge	Optimal viscosity, spreadability, retention time, permeation rate, and also high antifungal effect against dermatophytes.	[338]

Abbreviations: AgNPs—Silver nanoparticles, PVA—Polyvinyl alcohol, PVP—Polyvinylpyrrolidone.

**Table 8:** Recent studies on the use of hydroxypropylcellulose as a wound dressing.

Biopolymer/-s	Active Pharmaceutical Ingredient (Natural or Synthetic Substances)	Type of Wound Dressing	Main Findings	References
HPC	Peptide PXL150	Gel	Due to the antimicrobial activity, the gel healed the infection of third-degree burns injuries and surgical wounds.	[355]
	-	Hydrogel	Suitable water absorption; the hydrogel can maintain an adequate moisture balance at the lesion site.	[339]
	Graphene oxide, Isophorone diisocyanate, and Ag/ZnO	Film	Higher mechanical, anti-ultraviolet properties, and antimicrobial activity.	[356]
HPC/Gelatin	Chloramphenicol	Hydrogel	Adequate mechanical strength, water vapor permeability, light transmittance, and excellent antibacterial effect.	[357]
HPC/Chitosan/PEO	Graphene	Membrane	Proper mechanical properties, hydrophilicity, water vapor transmission rate, and high antibacterial effect; the membrane can impede the bacterial adhesion.	[341]
HPC/Sodium alginate	Gatifloxacin	Hydrogel	Advantageous physicochemical properties regarding the tensile strength, the swelling capacity, the elongation, and the drug release.	[236]
	Pioglitazone	Hydrogel	By adding HPC, the gelation behavior of the hydrogel increased, with an extended release of the drug up to 5 days; thus, the novel formulation can be used for skin ulcer treatment.	[358]

Abbreviations: PEO—polyethylene oxide, Ag—the chemical symbol of silver, ZnO—zinc oxide.

**Table 9:** Recent studies on the use of combinations of cellulose derivatives as wound dressing.

Biopolymer/-s	Active Pharmaceutical Ingredient (Natural or Synthetic Substances)	Type of Wound Dressing	Main Findings	References
EC/HPMC	Paromomycin and Gentamicin	Film	Optimum drugs release and inhibition of *Leishmania tropica growth.*	[359]
	Aloe vera	Nanofibers	Nanofibers with 10% Aloe vera showed suitable mechanical properties, biocompatibility, bioadhesion, and suitable antibacterial activity.	[360]
NaCMC/HPMC	Grapefruit seed extract	Film	Suitable elongation at break, stability in water, and proper antibacterial action.	[361]
	CuO	Film	Good biocompatibility and antibacterial effect.	[362]
	Tetracycline/Methylene blue	Film	Nanoporous network, increased T_g_ and elongation at break, sustained drug release for 72 h and high antibacterial effect.	[363]
	ZnO NPs	Film	Biocompatibility and optimum antibacterial action.	[364]
NaCMC/HPMC/ CAB	Resveratrol	Membrane	Excellent adhesive capacity, hydration efficiency, and higher porous structure; in vivo studies showed accelerated wound healing.	[365]
NaCMC/MC	Simvastatin	Membrane	In a ratio of 2:1, the membrane exhibited appropriate flexibility, viscosity, stability, and sponginess; optimal drug delivery for suppurating injuries.	[366]

Abbreviations: CAB—Cellulose acetate butyrate, CuO—Copper oxide, T_g_—Glass transition temperature, ZnO NPs—Zinc oxide nanoparticles.

## Data Availability

No new data were created or analyzed in this study. Data sharing is not applicable to this article.
